# Correction: Enzyme-powered micro- and nano-motors: key parameters for an application-oriented design

**DOI:** 10.1039/d2sc90162e

**Published:** 2022-08-11

**Authors:** Xavier Arqué, Tania Patiño, Samuel Sánchez

**Affiliations:** Institute for Bioengineering of Catalonia (IBEC), The Barcelona Institute of Science and Technology (BIST) Barcelona 08028 Spain ssanchez@ibecbarcelona.eu; Bio-Organic Chemistry, Institute for Complex Molecular Systems, Eindhoven University of Technology 5600 MB Eindhoven The Netherlands; Institució Catalana de Recerca i Estudis Avançats (ICREA) Barcelona 08010 Spain

## Abstract

Correction for ‘Enzyme-powered micro- and nano-motors: key parameters for an application-oriented design’ by Xavier Arqué *et al.*, *Chem. Sci.*, 2022, https://doi.org/10.1039/d2sc01806c.

The authors regret that several references were cited in incorrect locations in the figure captions. The correct captions are reproduced below.

**Fig. 1** Chassis materials of enzyme-powered micro- and nano-motors. (A) Representation of each chassis material in the publications of the field. Examples of enzymatic motors made of (B) polymers,^27,37,66^ (C) metals,^49,67,104^ (D) silica,^70,96^ (E) carbon,^107,108^ (F) lipid vesicles,^117,118^ (G) MOFs^112,115^ and (H) bio-inspired materials.^120–122^ Panel (B) adapted with permission from (1) ref. 27 Copyright 2019 Springer Nature, (2) ref. 37 Copyright 2017 AAAS, and (3) ref. 66 Copyright 2021 Royal Society of Chemistry. Panel (C) adapted with permission from (1) ref. 104 Copyright 2021 American Chemical Society, (2) ref. 49 Copyright 2019 American Chemical Society, and (3) ref. 67 Copyright 2019 Wiley. Panel (D) adapted with permission from (1) ref. 70 Copyright 2015 American Chemical Society and (2) ref. 96 Copyright 2021 AAAS. Panel (E) adapted with permission from (1) ref. 108 Copyright 2021 Wiley and (2) ref. 107 Copyright 2019 American Chemical Society. Panel (F) adapted with permission from (1) ref. 117 Copyright 2020 Wiley and (2) ref. 118 Copyright 2019 American Chemical Society. Panel (G) adapted with permission from (1) ref. 115 Copyright 2020 American Chemical Society and (2) ref. 112 Copyright 2019 Wiley. Panel (H) adapted with permission from (1) ref. 122 Copyright 2016 Wiley, (2) ref. 121 Copyright 2020 AAAS, and (3) ref. 120 Copyright 2022 American Chemical Society.

**Fig. 2** Chassis shape and product release distribution of enzyme-powered micro- and nano-motors. (A) Representation of each chassis shape in the publications of the field. Inset: representation of each configuration of product release in the publications of the field. Examples of enzymatic motors with shapes of (B) spheres,^15,29,62,69,89^ (C) tubes,^30,86^ (D) rods,^17,20,122^ (E) vesicles,^37,118^ (F) stomatocytes,^23,24,27^ (G) crystals,^112^ (H) bottles^35,98^ and (I) shells.^47^ Panel (B) adapted with permission from (1) ref. 15 Copyright 2019 Elsevier, (2) ref. 89 Copyright 2018 American Chemical Society, (3) ref. 29 Copyright 2019 Wiley, (4) ref. 69 Copyright 2017 Elsevier and (5) ref. 62 Copyright 2017 Royal Society of Chemistry. Panel (C) adapted with permission from (1) ref. 86 Copyright 2016 American Chemical Society and (2) ref. 30 Copyright 2016 Wiley. Panel (D) adapted with permission from (1) ref. 17 Copyright 2020 Elsevier, (2) ref. 122 Copyright 2016 Wiley, and (3) ref. 20 Copyright 2021 Elsevier. Panel (E) adapted with permission from (1) ref. 37 Copyright 2017 AAAS and (2) ref. 118 Copyright 2019 American Chemical Society. Panel (F) adapted with permission from (1) ref. 24 Copyright 2016 American Chemical Society, (2) ref. 27 Copyright 2019 Springer Nature and (3) ref. 23 Copyright 2016 American Chemical Society. Panel (G) adapted with permission from (1) ref. 112 Copyright 2019 Wiley. Panel (H) adapted with permission from (1) ref. 98 Copyright 2021 American Chemical Society and (2) ref. 35 Copyright 2022 Wiley. Panel (I) adapted with permission from (1) ref. 47 Copyright 2019 Wiley.

**Fig. 3** Enzyme incorporation method of enzyme-powered micro- and nano-motors. (A) Representation of each enzyme incorporation method in the publications of the field. Examples of enzymatic motors with enzymes attached by using covalent attachment like (B) EDC/NHS,^13,40,58,104^ (C) glutaraldehyde,^60,74,100^ (D) biotin/streptavidin^44,118,120^ or (E) other covalent methods,^21,27^ and non-covalent incorporation methods like (F) encapsulation^32,37,59,117^ and (G) non-covalent interaction.^30,35,65,74,76^ Panel (B) adapted with permission from (1) ref. 13 Copyright 2021 American Chemical Society, (2) ref. 58 Copyright 2017 Elsevier, (3) ref. 40 Copyright 2019 American Chemical Society, and (4) ref. 104 Copyright 2021 American Chemical Society. Panel (C) adapted with permission from (1) ref. 74 Copyright 2019 American Chemical Society, (2) ref. 100 Copyright 2021 American Institute of Physics and (3) ref. 60 Copyright 2021 American Chemical Society. Panel (D) adapted with permission from (1) ref. 120 Copyright 2020 AAAS, (2) ref. 118 Copyright 2019 American Chemical Society, and (3) ref. 44 Copyright 2020 American Chemical Society. Panel (E) adapted with permission from (1) ref. 27 Copyright 2019 Springer Nature and (2) ref. 21 Copyright 2022 Elsevier. Panel (F) adapted with permission from (1) ref. 59 Copyright 2015 American Chemical Society, (2) ref. 32 Copyright 2021 Multidisciplinary Digital Publishing Institute, (3) ref. 37 Copyright 2017 AAAS and (4) ref. 117 Copyright 2020 Wiley. Panel (G) adapted with permission from (1) ref. 76 Copyright 2020 Wiley, (2) ref. 30 Copyright 2016 Wiley, (3) ref. 65 Copyright 2019 American Chemical Society, (4) ref. 35 Copyright 2022 Wiley, and (5) ref. 74 Copyright 2019 American Chemical Society.

**Fig. 4** Enzyme type and motion mechanism of enzyme-powered micro- and nano-motors. (A) Representation of each enzyme type in the publications of the field. Inset: representation of each motion mechanism in the publications of the field. Examples of enzymatic motors powered with the enzymes (B) catalase,^38,43,45,54,61,98^ (C) urease,^20,22,39,44,82,100,120^ (D) glucose oxidase,^67,68^ (E) glucose oxidase and catalase,^27,35,37,91,95^ (F) lipase,^75,76^ (G) acetylcholinesterase,^83^ (H) trypsin,^103^ (I) enzyme combinations^52,105^ and (J) enzymatic pathway.^23^ Panel (B) adapted with permission from (1) ref. 54 Copyright 2013 American Chemical Society, (2) ref. 98 Copyright 2021 American Chemical Society, (3) ref. 61 Copyright 2019 American Chemical Society, (4) ref. 45 Copyright 2010 American Chemical Society, (5) ref. 38 Copyright 2018 American Chemical Society, and (6) ref. 43 Copyright 2019 Elsevier. Panel (C) adapted with permission from (1) ref. 120 Copyright 2020 AAAS, (2) ref. 22 Copyright 2020 American Chemical Society, (3) ref. 82 Copyright 2020 American Physical Society, (4) ref. 20 Copyright 2021 Elsevier, (5) ref. 39 Copyright 2019 Wiley, (6) ref. 44 Copyright 2020 American Chemical Society, and (7) ref. 100 Copyright 2021 American Institute of Physics. Panel (D) adapted with permission from (1) ref. 67 Copyright 2019 Wiley and (2) ref. 68 Copyright 2021 American Chemical Society. Panel (E) adapted with permission from (1) ref. 27 Copyright 2019 Springer Nature, (2) ref. 37 Copyright 2017 AAAS, (3) ref. 95 Copyright 2022 American Chemical Society, (4) ref. 91 Copyright 2015 American Chemical Society, and (5) ref. 35 Copyright 2022 Wiley. Panel (F) adapted with permission from (1) ref. 76 Copyright 2020 Wiley and (2) ref. 75 Copyright 2019 Wiley. Panel (G) adapted with permission from (1) ref. 83 Copyright 2019 Springer Nature and (2) ref. 70 Copyright 2015 American Chemical Society. Panel (H) adapted with permission from (1) ref. 103 Copyright 2017 American Chemical Society. Panel (I) adapted with permission from (1) ref. 52 Copyright 2014 Wiley and (2) ref. 105 Copyright 2005 American Chemical Society. Panel (J) adapted with permission from ref. 23 Copyright 2016 American Chemical Society.

**Fig. 5** (A) Sizes of enzyme-powered micro- and nano-motors and motion detection technique. Representation of the different motor sizes in the publications of the field. Inset: representation of each motion detection technique in the publications of the field. Examples of enzymatic motors powered with sizes of (B) <0.3 (ref. 69, 25, 104, 42, 37 and 98), (C) 0.3–1 (ref. 96, 31, 76, 79 and 107), (D and E) 1–10 (ref. 112, 111, 100, 84, 120, 62, 86, 17 and 54) and (F) >10 μm.^30,39,66,123^ Panel (B) <0.3 μm adapted with permission from ref. 69 Copyright 2017 Elsevier, ref. 25 Copyright 2019 American Chemical Society, ref. 104 Copyright 2021 American Chemical Society, ref. 42 Copyright 2021 Elsevier, ref. 37 Copyright 2017 AAAS, and ref. 98 Copyright 2021 American Chemical Society. Panel (C) 0.3–1 μm adapted with permission from ref. 96 Copyright 2021 AAAS, ref. 31 Copyright 2021 American Chemical Society, ref. 76 Copyright 2020 Wiley, ref. 79 Copyright 2021 American Chemical Society, and ref. 107 Copyright 2019 American Chemical Society. Panels (D and E) 1–10 μm adapted with permission from ref. 112 Copyright 2019 Wiley, ref. 111 Copyright 2021 American Chemical Society, ref. 100 Copyright 2021 American Institute of Physics, ref. 84 Copyright 2020 AAAS, ref. 120 Copyright 2020 AAAS, ref. 62 Copyright 2017 Royal Society of Chemistry, ref. 86 Copyright 2016 American Chemical Society, ref. 17 Copyright 2020 Elsevier, and ref. 54 Copyright 2013 American Chemical Society. Panel (F) >10 μm adapted with permission from ref. 66 Copyright 2021 Royal Society of Chemistry, ref. 39 Copyright 2019 Wiley, ref. 30 Copyright 2016 Wiley, and ref. 123 Copyright 2019 Wiley.

**Fig. 6** (A) Applications of enzyme-powered micro- and nano-motors and motion detection technique. Representation of the different motor applications in the publications of the field. Inset: representation of each motion media in the publications of the field. Examples of enzymatic motors applied in (B) drug delivery treatment,^20,21,42,59,111,121^ (C) visualization inside organisms exploiting enhanced targeting and penetration,^25,40,62,73,96,104^ (D) photo- and magnetic thermal treatment,^65,113^ (E) cell and compound sensing,^17,43,49,58,81^ (F) contaminant compound removal^13,75,115^ and (G) bacteria capture or elimination.^60,66,72,78^ Panel (B) adapted with permission from (1) ref. 121 Copyright 2022 American Chemical Society, (2) ref. 42 Copyright 2021 Elsevier, (3) ref. 21 Copyright 2022 Elsevier, (4) ref. 20 Copyright 2021 Elsevier, (5) ref. 59 Copyright 2015 American Chemical Society, and (6) ref. 111 Copyright 2021 American Chemical Society. Panel (C) adapted with permission from (1) ref. 104 Copyright 2021 American Chemical Society, (2) ref. 73 Copyright 2019 American Chemical Society, (3) ref. 40 Copyright 2019 American Chemical Society, (4) ref. 96 Copyright 2021 AAAS, (5) ref. 62 Copyright 2017 Royal Society of Chemistry, and (6) ref. 25 Copyright 2019 American Chemical Society. Panel (D) adapted with permission from (1) ref. 113 Copyright 2019 Elsevier and (2) ref. 65 Copyright 2019 American Chemical Society. Panel (E) adapted with permission from (1) ref. 43 Copyright 2019 Elsevier, (2) ref. 49 Copyright 2019 American Chemical Society, (3) ref. 17 Copyright 2020 Elsevier, (4) ref. 58 Copyright 2017 Elsevier, and (5) ref. 81 Copyright 2019 American Chemical Society. Panel (F) adapted with permission from (1) ref. 13 Copyright 2021 American Chemical Society, (2) ref. 75 Copyright 2019 Wiley, and (3) ref. 115 Copyright 2020 American Chemical Society. Panel (G) adapted with permission from (1) ref. 60 Copyright 2021 American Chemical Society, (2) ref. 66 Copyright 2021 Royal Society of Chemistry, (3) ref. 72 Copyright 2021 American Chemical Society, and (4) ref. 78 Copyright 2022 American Chemical Society.

The Royal Society of Chemistry apologises for these errors and any consequent inconvenience to authors and readers.

## Supplementary Material

